# Jarisch-Herxheimer reaction among HIV-positive patients with early syphilis: azithromycin versus benzathine penicillin G therapy

**DOI:** 10.7448/IAS.17.1.18993

**Published:** 2014-08-28

**Authors:** Mao-Song Tsai, Chia-Jui Yang, Nan-Yao Lee, Szu-Min Hsieh, Yu-Hui Lin, Hsin-Yun Sun, Wang-Huei Sheng, Kuan-Yeh Lee, Shan-Ping Yang, Wen-Chun Liu, Pei-Ying Wu, Wen-Chien Ko, Chien-Ching Hung

**Affiliations:** 1 Department of Internal Medicine, Far Eastern Memorial Hospital, New Taipei City, Taiwan; 2 Department of Internal Medicine, National Cheng Kung University College of Medicine and Hospital, Tainan, Taiwan; 3 Department of Internal Medicine, National Taiwan University Hospital and National Taiwan University College of Medicine, Taipei, Taiwan; 4 Department of Internal Medicine, Taichung Veterans General Hospital, Taichung, Taiwan; 5 Department of Internal Medicine, National Taiwan University Hospital Hsin-Chu Branch, Hsin-Chu, Taiwan; 6 Center for Infection Control, National Taiwan University Hospital, Taipei, Taiwan; 7 Department of Medical Research, China Medical University Hospital, Taichung, Taiwan; 8 China Medical University, Taichung, Taiwan

**Keywords:** sexually transmitted diseases, spirochetal disease, macrolides, macrolide resistance, immunomodulation

## Abstract

**Introduction:**

The Jarisch-Herxheimer reaction, a febrile inflammatory reaction that often occurs after the first dose of chemotherapy in spirochetal diseases, may result in deleterious effects to patients with neurosyphilis and to pregnant women. A single 2-g oral dose of azithromycin is an alternative treatment to benzathine penicillin G for early syphilis in areas with low macrolide resistance. With its potential anti-inflammatory activity, the impact of azithromycin on the incidence of the Jarisch-Herxheimer reaction in HIV-positive patients with early syphilis has rarely been investigated.

**Methods:**

In HIV-positive patients with early syphilis, the Jarisch-Herxheimer reaction was prospectively investigated using the same data collection form in 119 patients who received benzathine penicillin G between 2007 and 2009 and 198 who received azithromycin between 2012 and 2013, when shortage of benzathine penicillin G occurred in Taiwan. Between 2012 and 2013, polymerase chain reaction (PCR) assay was performed to detect *Treponema pallidum* DNA in clinical specimens, and PCR restriction fragment length polymorphism of the 23S ribosomal RNA was performed to detect point mutations (2058G or A2059G) that are associated with macrolide resistance.

**Results:**

The overall incidence of the Jarisch-Herxheimer reaction was significantly lower in patients receiving azithromycin than those receiving benzathine penicillin G (14.1% vs. 56.3%, *p<*0.001). The risk increased with higher rapid plasma reagin (RPR) titres (adjusted odds ratio [AOR] per 1-log_2_ increase, 1.21; confidence interval [CI], 1.04–1.41), but decreased with prior penicillin therapy for syphilis (AOR, 0.37; 95% CI, 0.19–0.71) and azithromycin treatment (AOR, 0.15; 95% CI, 0.08–0.29). During the study period, 310 specimens were obtained from 198 patients with syphilis for PCR assays, from whom *T. pallidum* was identified in 76 patients, one of whom (1.3%) was found to be infected with *T. pallidum* harbouring the macrolide resistance mutation (A2058G). In subgroup analyses confined to the 75 patients infected with *T. pallidum* lacking resistance mutation, a statistically significantly lower risk for the Jarisch-Herxheimer reaction following azithromycin treatment was noted.

**Conclusions:**

Treatment with azithromycin was associated with a lower risk for the Jarisch-Herxheimer reaction than that with benzathine penicillin G in HIV-positive patients with early syphilis. Previous benzathine penicillin G therapy for syphilis decreased the risk, whereas higher RPR titres increased the risk, for the reaction.

## Introduction

According to the Sexually Transmitted Diseases (STDs) Treatment Guidelines 2010 by the US Centers for Disease Control and Prevention (CDC) [1], a single dose of benzathine penicillin G is recommended for patients with early syphilis, and three weekly doses of benzathine penicillin G are recommended for those with late latent syphilis or syphilis of unknown duration. In patients who are intolerant of penicillin, azithromycin as a single 2-g oral dose can be an alternative in areas of low prevalence of *Treponema pallidum* with macrolide resistance [2–4].

The Jarisch-Herxheimer reaction is a febrile inflammatory reaction that often occurs after the first dose of chemotherapy in spirochetal diseases, with common clinical manifestations that include fever, rigours, sweats, hypotension and worsening of skin rashes[5–12]. The incidence of the Jarisch-Herxheimer reaction varies among the published studies, depending on the stage of syphilis and the patient populations studied. For example, 50 to 75% of the patients with primary or secondary syphilis may experience the Jarisch-Herxheimer reaction, which typically occurs within 2 to 5 hours of initiation of penicillin therapy for syphilis and usually resolves within 24 hours [7,13,14]. In the study by Miller *et al*., the overall incidence is around 9% in syphilis patients regardless of stage [15]. Patients with HIV infection and pregnant women have a higher incidence of the Jarisch-Herxheimer reaction while receiving syphilis treatment, with a rate of 35 and 40%, respectively [16,17]. While most of the cases of the Jarisch-Herxheimer reaction are self-limited, it may result in potentially life-threatening consequences. The Jarisch-Herxheimer reaction in pregnant women has been associated with early spontaneous termination of pregnancy and premature labour when penicillins, the only safe and effective agent for the treatment of syphilis in pregnancy, are administered [17– 19]. The reaction can result in acute deleterious effects, such as central nervous system inflammation associated with elevations of several cytokines [6,20,21]. Agents that may help prevent or ameliorate the Jarisch-Herxheimer reaction are either not available in routine practice or not entirely effective [21–26].

Azithromycin is an azalide antibiotic that exerts effect through inhibition of protein synthesis with a long half-life in tissue (48–96 hours) and a slow release of drug from the tissue followed by elimination from the vascular compartment. It has been observed to exert anti-inflammatory actions through modulation of cytokine release independent of its antimicrobial properties [27–29]. In this quasi-experimental, two-sample study, we aimed to compare the incidence of the Jarisch-Herxheimer reaction following azithromycin versus benzathine penicillin G treatment in HIV-positive patients with early syphilis. We hypothesized that the incidence of the Jarisch-Herxheimer reaction in HIV-positive patients with early syphilis who received azithromycin was lower than that of those who received benzathine penicillin G.

## Methods

### Study population and setting

This was a non-concurrent, two-sample cohort study of the incidence and risk factors of the Jarisch-Herxheimer reaction in HIV-positive patients with early syphilis who received treatment with benzathine penicillin G or azithromycin. In the late spring of 2012, a shortage of benzathine penicillin G occurred in Taiwan. Based on our previous surveillance study showing a lack (0%) of significant macrolide-resistant mutations (A2058G or A2059G) among 105 strains of *T. pallidum* [30] and the randomized controlled trial in Africa that demonstrated the non-inferiority of azithromycin to benzathine penicillin G in treatment of early syphilis [2], a single 2-gram dose of azithromycin (250 mg/tablet, Zithromax; Pfizer, Groton, CT, USA) was used as a substitute for benzathine penicillin G to treat HIV-positive patients with early syphilis between May 2012 and July 2013. HIV-positive patients receiving benzathine penicillin G for early syphilis, who were enrolled in a multicentre, prospective observational study of the Jarisch-Herxheimer reaction between January 2007 and December 2009 [16], were included as a comparator group.

HIV-positive patients with syphilis received a counselling session to reduce risks for recurrent STDs according to the HIV case management programme that was implemented by the Taiwan Centers for Disease Control as public health responses to control HIV infection and STDs. Patients with STDs are invited to participate in an HIV case management programme. Treatment and follow-up of syphilis are integral components of the programme. The study was approved by the Research Ethics Committee of each hospital, and participants gave written informed consent.

### Diagnosis, staging and treatment for syphilis

Syphilis is a reportable infectious disease in Taiwan, for which the diagnostic criterion is a titre of ≥1:320 by *T. pallidum* particle agglutination (TPPA) assays (SERODIA-TPPA; Fujirebio, Tokyo, Japan). In this study, diagnosis of syphilis was made based on a positive TPPA reaction and a titre of rapid plasma reagin (RPR) ≥1:4 (RPR Card test; Becton-Dickinson, Sparks, MD, USA) to reduce the risk of a false-positive reaction in HIV-positive patients and to facilitate the assessment of treatment response. Early syphilis included primary, secondary and early latent syphilis [1]. Patients were diagnosed as having primary syphilis if they had ulceration of the anogenital or oral region (chancre); secondary syphilis was defined as the presence of a cutaneous rash, mucosal lesions, generalized lymphadenopathy or other signs; and early latent syphilis was defined as syphilis that was acquired within the preceding year and characterized by seroreactivity without other evidence of disease.

Azithromycin was administered under the direct supervision of HIV case managers after the patient took a light meal to alleviate the gastrointestinal adverse effects. A cell phone call to the patients was made by the case managers within 24 hours of azithromycin or penicillin therapy to inquire about the reaction after treatment, and all the information was collected in a standardized data collection form. The Jarisch-Herxheimer reaction was defined as the presence of fever >38.0°C taken using an electronic thermometer by the patients and/or acute exacerbation of maculopapular skin rashes within 24 hours of receipt of antibiotic therapy for syphilis. Photos were taken using cell phones before treatment in patients with syphilis-related skin rashes for comparison with those taken when the Jarisch-Herxheimer reaction developed.

### Investigation of azithromycin-resistant *T. pallidum*

After August 2010, all patients seeking syphilis treatment at the National Taiwan University Hospital were invited to participate in a surveillance study of *T. pallidum* with macrolide resistance [31]. Serum and plasma samples were obtained from patients with early syphilis using anticoagulant-free and EDTA-coated containers (BD Vacutainer tubes; Becton Dickinson, Plymouth, UK), respectively. Sampling of multiple specimens was performed when patients presented with multiple ulcers at different affected sites in cases of primary and secondary lesions. Specimens of chancre lesions were collected with the use of swabs that were pressed and rolled over the ulcers. Lesion exudate collected with a swab (CultureSwab EZ; BD, Franklin Lakes, NJ, USA) was immediately transported to the research laboratory at the National Taiwan University Hospital. The swab was subsequently placed in 1 ml of sterile phosphate-buffered saline (PBS) at 4°C overnight, which was centrifuged for 10 min at 13,000×g at room temperature in order to obtain the cell pellet corresponding to treponemal DNA. All samples were stored at −80°C until extraction. Treponemal DNA was extracted with the QIAamp DNA minikit (Qiagen GmbH, Düsseldorf, Germany) according to the manufacturer's instructions. Polymerase chain reaction (PCR) assay was performed to detect the presence of *T. pallidum* by amplifying a 378-bp fragment of the *T. pallidum* polymerase A gene (*pol*A) [32]. *T. pallidum* harbouring an A2058G or A2059G point mutation [33,34] was investigated using restriction fragment length polymorphism (RFLP) [35]. In brief, the 23S ribosomal RNA (rRNA) gene of *T. pallidum* was amplified and subjected to MboII and BsaI digestion (New England Biolabs, Beverly, MA, USA). The mutations were confirmed by comparison with previous studies after gel electrophoresis [33,35]. In this study, only those patients who received azithromycin and underwent resistance testing were included for analysis; the comparator group who received penicillin were enrolled between 2007 and 2009, before the onset of research laboratory testing for macrolide resistance mutations.

### Statistical analysis

The analyses were conducted using the statistical package SAS 9.3 (SAS Institute Inc., Cary, NC, USA). Chi-square tests – or, if necessary, Fisher's exact tests – were used for categorical variables. Student's *t* and Mann-Whitney U tests were used for numerical variables. A normal approximation was used in calculating the confidence interval (CI) that was set at 95%. Logistic regression analysis was used to identify factors associated with the Jarisch-Herxheimer reaction. The regression models were built using a forward stepwise procedure using demographic characteristics, clinical characteristics that included stage of syphilis, an RPR titre before treatment, prior treatment for syphilis, treatment regimen, and HIV status that included receipt of combination antiretroviral therapy, virological response to combination antiretroviral therapy and CD4 counts that were chosen *a priori* on the basis of hypotheses regarding factors that may affect response. All statistical tests were two-tailed, and *p* values<0.05 were considered to be statistically significant.

## Results

During the study period, 317 HIV-positive patients with early syphilis were enrolled: 119 (37.5%) received benzathine penicillin G between January 2007 and December 2009, and 198 (62.4%) received azithromycin between May 2012 and July 2013. The clinical characteristics of the patients are shown in Table 1. The mean age of the patients was 33.1 years, and 99.7% (*n=*316) were male. Compared with patients receiving benzathine penicillin G, patients receiving azithromycin had a higher CD4 count; were more likely to be on combination antiretroviral therapy (81.8% vs. 56.3%; *p<*0.001) with good viral suppression (plasma HIV RNA load <50 copies/mL) when early syphilis was diagnosed, to have received a previous diagnosis of syphilis and to present with early syphilis, and they were less likely to have an RPR titre ≥32.

**Table 1:** Clinical characteristics of HIV-positive patients with early syphilis who received benzathine penicillin G or azithromycin

Characteristics	Benzathine penicillin G, *n=*119	Azithromycin, *N=*198	*p*
Age, mean (SD), years	32.7 (8.7)	33.4 (7.5)	0.48
Male gender, *n* (%)	119 (100)	197 (99.5)	>0.99
MSM, *n* (%)	117 (98.3)	195 (98.0)	0.83
CD4 count, mean (SD), cells/µL	396.4 (239.4)	544.6 (246.6)	<0.001
Plasma HIV RNA load <50 copies/mL, *n* (%)	26 (21.8)	127 (64.1)	<0.001
Receipt of cART at the diagnosis of syphilis, *n* (%)	67 (56.3)	162 (81.8)	<0.001
Prior penicillin therapy for syphilis, *n* (%)	29 (24.3)	138 (69.7)	<0.001
Median RPR titre (IQR)	1:64 (32–128)	1:64 (16–128)	0.15
RPR ≥1:32, *n* (%)	99 (83.1)	144 (72.7)	0.03
Stage of syphilis, *n* (%)			<0.001
Primary	13 (10.9)	35 (17.7)	
Secondary	105 (88.2)	60 (30.3)	
Early latent	1 (0.8)	103 (52.0)	
Jarisch-Herxheimer reaction, *n* (%)	67 (56.3)	28 (14.1)	<0.001
Onset of Jarisch-Herxheimer reaction following treatment (IQR), hours	4 (3–6)	8 (5–19)	0.012

cART=combination antiretroviral therapy; IQR=interquartile range; MSM=men who have sex with men; SD=standard deviation; RPR=rapid plasma reagin.

**Figure 1:**
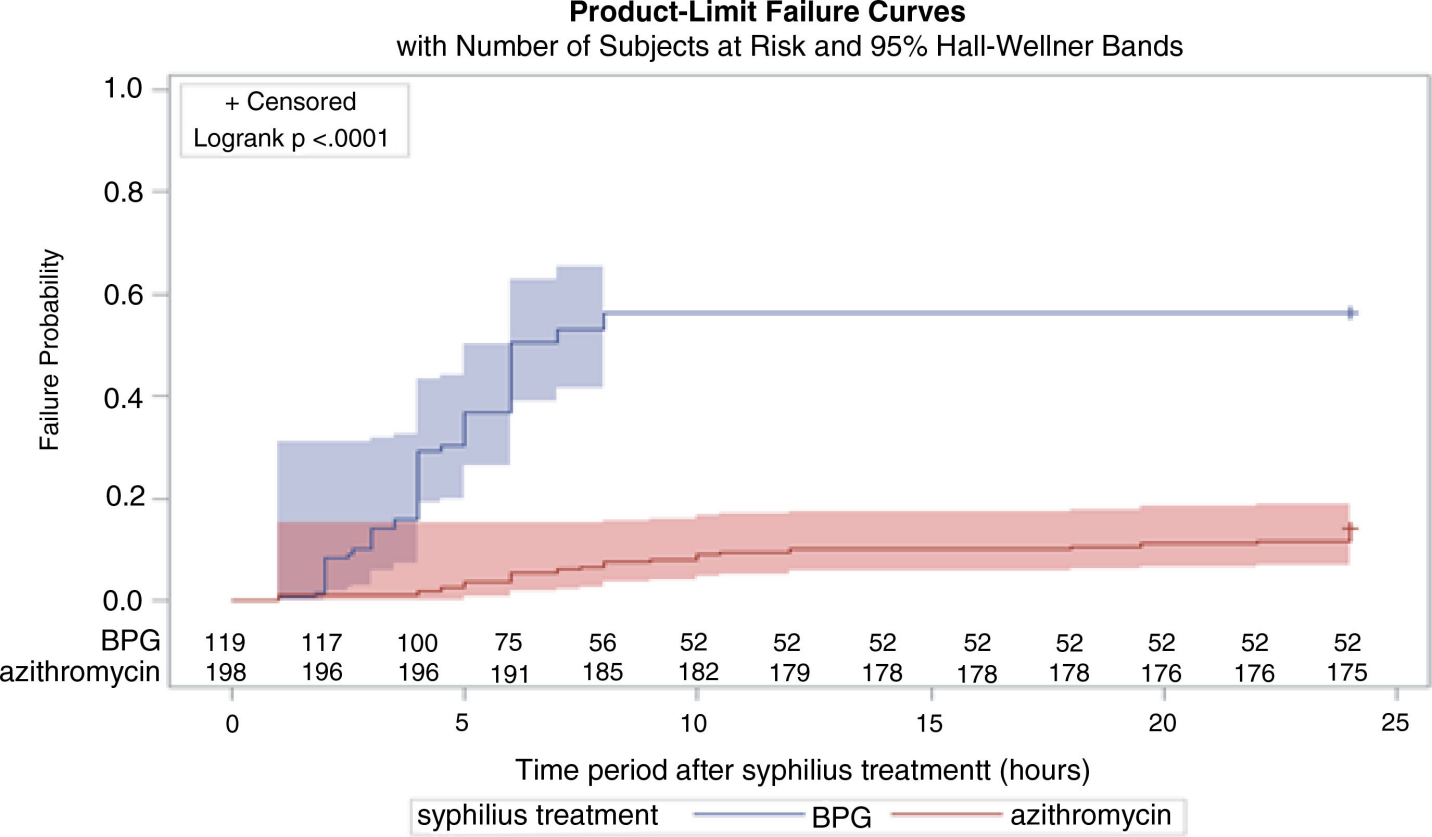
Time to the Jarisch-Herxheimer reaction during the first 24 hours after starting syphilis treatment according to the regimen (BPG, benzathine penicillin G).

The overall incidence of the Jarisch-Herxheimer reaction was 30.0% (95% CI, 25.2–35.2). Patients receiving benzathine penicillin G experienced a higher incidence of the Jarisch-Herxheimer reaction than those receiving azithromycin (56.3% vs. 14.1%; odds ratio [OR], 3.98; 95% CI, 2.73–5.81). The median time between treatment administration and development of the Jarisch-Herxheimer reaction was 4 hours [interquartile range (IQR), 3 to 6 hours] for the penicillin group, which was significantly shorter (8 hours) than that for the azithromycin group (IQR, 5–19 hours, *p<*0.001) (Figure 1). The symptoms such as fever or skin rash subsided spontaneously or with antipyretics and antihistamines within 24 hours.

In multivariate logistic regression, prior penicillin therapy for syphilis (adjusted odds ratio [AOR], 0.37; 95% CI, 0.19–0.71; *p=*0.003) and azithromycin versus benzathine penicillin G therapy (AOR, 0.15; 95% CI, 0.08–0.29; *p<*0.001) were two independent protective factors for the Jarisch-Herxheimer reaction. A statistically significant dose-response relationship was observed between RPR titre and the Jarisch-Herxheimer reaction (per 1-log_2_ RPR increase, AOR, 1.21; 95% CI, 1.04–1.41; *p=*0.013). We found no associations between the Jarisch-Herxheimer reaction and age, CD4 cell count and plasma HIV RNA load at the diagnosis of early syphilis or the receipt of combination antiretroviral therapy (Table 2).

**Table 2:** AORs and 95% CIs for factors associated with development of the Jarisch-Herxheimer reaction in multivariate analysis of different subgroups

All HIV-positive patients with early syphilis (*n=*317)				

Variables	Reference	AOR	95% CI	*p*
Age, years	Per 1-year increase	0.98	0.95–1.02	0.32
CART	Without cART	1.60	0.74–3.46	0.24
CD4 count, cells/µl	Per 100-cell/µl increase	0.99	0.88–1.12	0.84
PVL<50 copies/mL	PVL ≥50 copies/mL	1.14	0.50–2.58	0.76
Prior penicillin therapy for syphilis	No prior penicillin therapy for syphilis	0.37	0.19–0.71	0.003
RPR titres	Per 1-log_2_ RPR increase	1.21	1.04–1.41	0.013
Azithromycin	Benzathine penicillin G	0.15	0.08–0.29	<0.001
**Patients with primary or secondary syphilis (** ***N=*** **213)**				
Age, years	Per 1-year increase	0.98	0.94–1.02	0.37
CART	Without cART	1.46	0.67–3.21	0.34
CD4 count, cells/µl	Per 100-cell/µl increase	1.02	0.89–1.18	0.74
PVL<50 copies/mL	PVL ≥50 copies/mL	0.94	0.39–2.26	0.88
Prior penicillin therapy for syphilis	No prior penicillin therapy for syphilis	0.46	0.23–0.94	0.03
RPR titres	Per 1-log_2_ RPR increase	1.15	0.97–1.36	0.097
Azithromycin	Benzathine penicillin G	0.28	0.14–0.56	<0.001
**HIV-positive patients without prior syphilis treatment (** ***N=*** **150)**				
Age, years	Per 1-year increase	0.98	0.94–1.03	0.49
CART	Without cART	1.92	0.78–4.73	0.16
CD4 count, cells/µl	Per 100-cell/µl increase	1.17	0.96–1.43	0.12
PVL<50 copies/mL	PVL ≥50 copies/mL	0.91	0.30–2.74	0.86
RPR titres	Per 1-log_2_ RPR increase	1.16	0.91–1.36	0.28
Azithromycin	Benzathine penicillin G	0.12	0.05–0.30	<0.001

CART=combination antiretroviral therapy; AOR=adjusted odds ratio; PVL=plasma HIV RNA load; RPR=rapid plasma reagin; CI=confidence interval.

When we limited the analyses to those with primary (*n=*48) or secondary (*n=*165) syphilis, we found that 44.4% of the patients experienced the Jarisch-Herxheimer reaction. In multivariate logistic regression, prior penicillin therapy for syphilis (AOR, 0.46; 95% CI, 0.23–0.94; *p=*0.03) and azithromycin (AOR, 0.28; 95% CI, 0.14–0.56; *p<*0.001) remained independent protective factors of the Jarisch-Herxheimer reaction, while the RPR titre was not statistically significantly associated with the Jarisch-Herxheimer reaction (per 1-log_2_ RPR increase, AOR, 1.15; 95% CI, 0.97–1.36; *p=*0.097) (Table 2).

When we analyzed only the patients without a prior history of syphilis treatment (*n=*150), azithromycin compared with benzathine penicillin G (AOR, 0.12; 95% CI, 0.05–0.30; *p<*0.001) remained the only independent protective factor of the Jarisch-Herxheimer reaction (Table 2).

To mitigate the bias related to ineffective treatment for *T. pallidum*, a planned subgroup analysis was conducted. During the study period, a total of 310 specimens were collected from 198 patients, with multiple types of samples from 105 patients (226 specimens), and 76 patients (38.4%) had *T. pallidum* DNA identified in the clinical samples by PCR assays. *T. pallidum* DNA was detected in 21.0% (22/105) and 38.8% (47/121) of plasma samples and swab samples of chancre, respectively. One of 76 patients (1.3%) had a clinical strain of *T. pallidum* that was tested positive for the A2058G mutation. Therefore, we focused on the remaining 75 patients without known macrolide-resistant *T. pallidum* who received azithromycin. A significantly higher rate of the Jarisch-Herxheimer reaction was seen in patients receiving benzathine penicillin G than those receiving azithromycin (56.3% vs. 23.0%; OR, 2.45; 95% CI, 1.57–3.83). In multivariate logistic regression, a lower risk for the Jarisch-Herxheimer reaction following azithromycin treatment was consistently noted for all of the subgroup analyses (Table 3).

**Table 3:** Planned subgroup analysis for factors associated with the Jarisch-Herxheimer reaction in patents infected with *Treponema pallidum* without macrolide resistance mutations

Patients with early syphilis (*n=*194)				

Variables	Reference	AOR	95% CI	*p*
Age, years	Per 1-year increase	0.99	0.95–1.03	0.49
CART	Without cART	1.71	0.75–3.92	0.20
CD4 count, cells/µl	Per 100-cell/µl increase	1.05	0.90–1.22	0.55
PVL<50 copies/mL	PVL ≥50 copies/mL	0.98	0.38–2.54	0.97
Prior penicillin therapy for syphilis	No prior penicillin therapy for syphilis	0.43	0.21–0.90	0.03
RPR titres	Per 1-log_2_ RPR increase	1.17	0.99–1.39	0.07
Azithromycin	Benzathine penicillin G	0.21	0.10–0.48	<0.001
**Patients with primary or secondary syphilis (** ***N=*** **168)**				
Age, years	Per 1-year increase	0.99	0.95–1.03	0.55
CART	Without cART	1.62	0.71–3.72	0.26
CD4 count, cells/µl	Per 100-cell/µl increase	1.05	0.90–1.23	0.51
PVL<50 copies/mL	PVL≥50 copies/mL	0.92	0.35–2.44	0.87
Prior penicillin therapy for syphilis	Prior penicillin therapy for syphilis	0.51	0.23–1.09	0.08
RPR titres	Per 1-log_2_ RPR increase	1.15	0.97–1.37	0.11
Azithromycin	Benzathine penicillin G	0.32	0.14–0.75	0.008
**Patients without prior syphilis treatment (** ***N=*** **118)**				
Age, years	Per 1-year increase	0.99	0.94–1.04	0.72
CART	Without cART	2.08	0.78–5.56	0.14
CD4 count, cells/µl	Per 100-cell/µl increase	1.29	1.00–1.66	0.05
PVL<50 copies/mL	PVL≥50 copies/mL	1.36	0.38–4.88	0.63
RPR titres	Per 1-log_2_ RPR increase	1.14	0.91–1.43	0.25
Azithromycin	Benzathine penicillin G	0.11	0.03–0.36	<0.001

CART=combination antiretroviral therapy; AOR=adjusted odds ratio; PVL=plasma HIV RNA load; RPR=rapid plasma reagin; CI=confidence interval.

**Figure 2:**
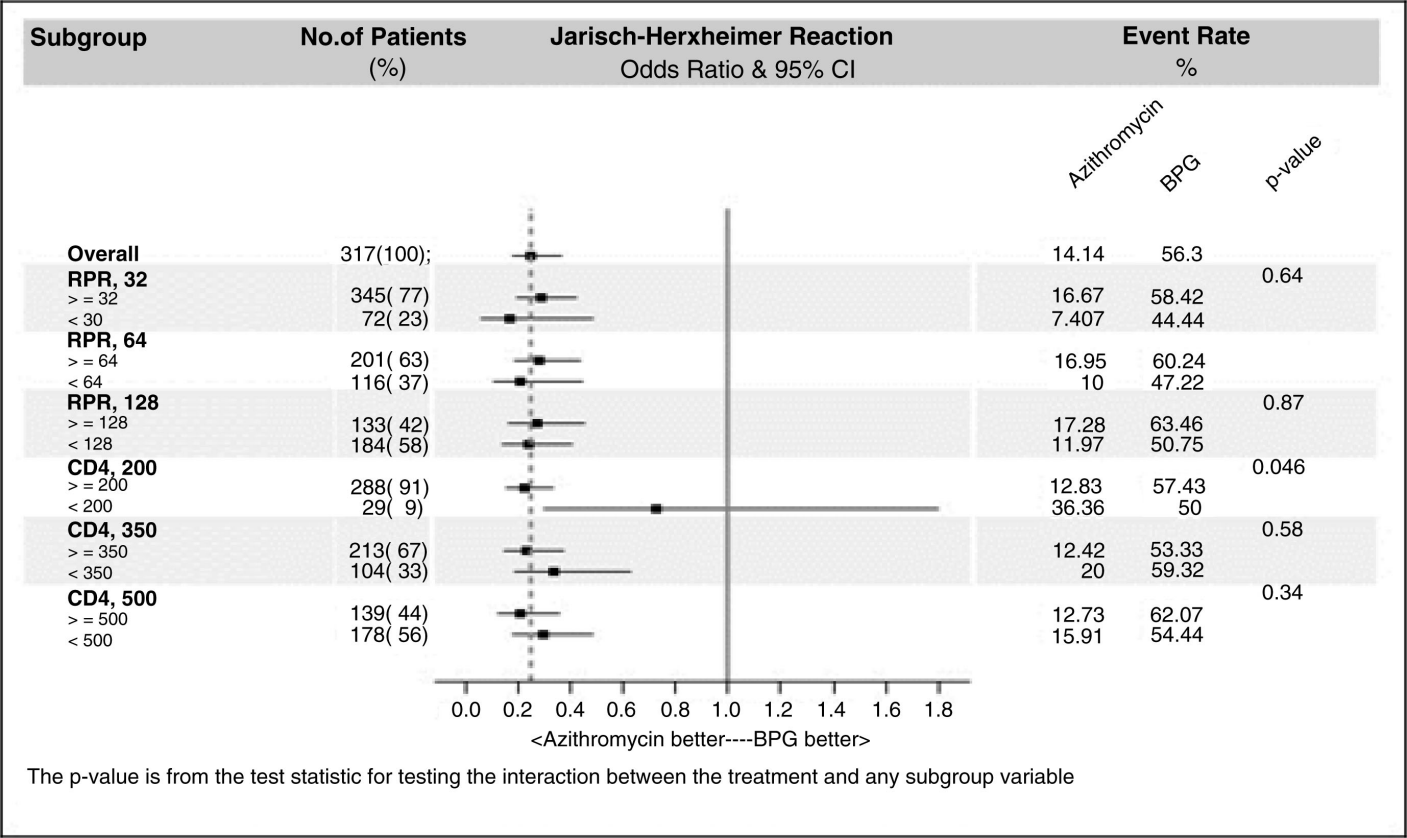
Forest plot showing the risk of the Jarisch-Herxheimer reaction according to subgroups (BPG, benzathine penicillin G; RPR, rapid plasma reagin).

The results of subgroup analyses are plotted to assess the magnitude of benefit across subgroups defined according to RPR titre and CD4 count with different cut-off values (Figure 2). Regardless of the different cut-off values used in the analysis, patients receiving azithromycin consistently demonstrated a lower risk of Jarisch-Herxheimer reaction than those receiving benzathine penicillin G.

## Discussion

In this prospective observational study, we found that azithromycin reduced the risk of the Jarisch-Herxheimer reaction by 80% or more when compared with benzathine penicillin G in treatment of HIV-positive patients with early syphilis; furthermore, the onset of the Jarisch-Herxheimer reaction in patients receiving azithromycin rather than benzathine penicillin G was significantly delayed (Figure 1).

Similar to our previous report [16], the risk of the Jarisch-Herxheimer reaction increased by 21% per 1-log_2_ increase of RPR titre and decreased by 63% with prior penicillin therapy for syphilis, independent of treatment administered. To our knowledge, there was no direct evidence supporting the association between RPR titre and spirochetal load. However, the findings that high RPR titres at treatment and during delivery in pregnant women are associated with an increased risk of delivery of a congenitally infected neonate after adequate treatment for maternal syphilis imply the association [18]. These findings suggest that a higher load of spirochetes in the early stages of syphilis increases the risk of the Jarisch-Herxheimer reaction when patients receive bactericidal agents. But the explanation of why prior penicillin therapy for syphilis is related to decreased frequency of the Jarisch-Herxheimer reaction is not clear.

The Jarisch-Herxheimer reaction can occur with many medications, including tetracyclines, penicillins, bismuth and sulphonamides, as long as the anti-treponemal concentrations are sufficient [36]. In this study, we found that azithromycin was consistently associated with decreased risk of the Jarisch-Herxheimer reaction compared with benzathine penicillin G in six different analyses. While the mechanisms for this finding warrant further investigation, the differential risk of the Jarisch-Herxheimer reaction between azithromycin and benzathine penicillin G is apparent clinically.

A number of plausible mechanisms exist to explain the discrepant incidence of the Jarisch-Herxheimer reaction between azithromycin and benzathine penicillin G. Benzathine penicillin G administered intramuscularly will reach peak concentration faster than azithromycin administered orally, which may contribute to the finding that the interval between treatment administration and onset of the Jarisch-Herxheimer reaction was shorter for benzathine penicillin G (4 hours) than for azithromycin (8 hours) (Figure 1). In the rabbit model, the median time to negativity by dark-field microscopy was longer in animals given azithromycin compared with animals given benzathine penicillin G, reinforcing the hypothesis [37].

Another widely held theory is that the destruction of spirochetes upon anti-treponemal treatment could activate the cytokine cascade and release of lipoproteins [5]. Penicillin treatment through inhibition of bacterial cell wall synthesis makes spirochetes more susceptible to phagocytosis, which then stimulates cytokine release, including tumour necrosis factor (TNF) and interleukins 6 and 8 (IL-6 and IL-8, respectively). Attempts at modulating the inflammatory response to anti-treponemal treatment may alleviate the risk [21]. Azithromycin exerts bactericidal effects through inhibition of protein synthesis. Furthermore, macrolides such as azithromycin have long been recognized to exert immunomodulary and anti-inflammatory actions [38]. Increasing data suggest that azithromycin suppresses the production of inflammatory cytokines such as TNF-*α*, IL-1*β*, IL-6 and macrophage inflammatory protein 2 apart from the antibiotic effect not only in respiratory diseases but also in genital infections [38,39].

There are many compelling reasons for performing the subgroup analyses. Despite the earlier finding in Taiwan that none of the 105 patients sampled carried *T. pallidum* strains with the macrolide resistance mutations between 2009 and 2011 [30], one of the major public health concerns is the emergence of azithromycin-resistant syphilis in several countries [40–44], which may lead to treatment failures and confound our observation of the Jarisch-Herxheimer reaction [45]. In this study, the prevalence of *T. pallidum* harbouring the macrolide resistance mutation remains low (1.3%). Our causal subgroup analyses, which consistently demonstrated a lower incidence of the Jarisch-Herxheimer reaction following treatment with azithromycin compared to benzathine penicillin G in the patients infected with *T. pallidum* without harbouring the macrolide resistance mutation, may help minimize the bias and draw a robust conclusion.

Our study is limited by the fact that the study was conducted in two different time periods when treatment for early syphilis was affected by the shortage of benzathine penicillin G. Secondly, the definitions for the Jarisch-Herxheimer reaction are non-specific without clinically applicable surrogate biomarkers, for which the diagnosis is made clinically on the basis of the presentations and timing of therapy for syphilis is administered. In this study, we encouraged the patients to use an electronic thermometer and cell phone to facilitate the detection of the reaction. Although we used the same definition for the Jarisch-Herxheimer reaction and the same case record form to prospectively collect the symptoms following treatment in order to minimize the bias in this two-sample cohort study, our results need to be externally validated by assessing their applicability to data collected by different investigators. Thirdly, because of concerns about an increased risk of biologic false-positive syphilis serologies (RPR titre of 1:1 or 1:2) [46], we decided to choose 1:4 or greater as a cut-off [16]. It is therefore unclear if patients with RPR titres <4 would develop the Jarisch-Herxheimer reaction. Lastly, the observation duration is suitable only for survey of the Jarisch-Herxheimer reaction but is insufficient to assess treatment effectiveness for different treatment regimens.

In conclusion, azithromycin therapy and previous penicillin therapy for syphilis may reduce the risk for the Jarisch-Herxheimer reaction in HIV-positive patients with early syphilis, whereas higher RPR titres increase the risk for the reaction. The results may have relevance for the management of early syphilis and the investigations of innate inflammatory response associated with syphilis.
